# Influence of Various Polymorphic Variants of Cytochrome P450 Oxidoreductase (POR) on Drug Metabolic Activity of CYP3A4 and CYP2B6

**DOI:** 10.1371/journal.pone.0038495

**Published:** 2012-06-12

**Authors:** Xuan Chen, Li Qiang Pan, Hua Naranmandura, Su Zeng, Shu Qing Chen

**Affiliations:** 1 Department of Pharmacology, Toxicology and Biochemical Pharmaceutics, College of Pharmaceutical Sciences, Zhejiang University, Hangzhou, China; 2 Department of Drug Metabolism and Pharmaceutical Analysis, College of Pharmaceutical Sciences, Zhejiang University, Hangzhou, China; Concordia University Wisconsin, United States of America

## Abstract

Cytochrome P450 oxidoreductase (POR) is known as the sole electron donor in the metabolism of drugs by cytochrome P450 (CYP) enzymes in human. However, little is known about the effect of polymorphic variants of POR on drug metabolic activities of CYP3A4 and CYP2B6. In order to better understand the mechanism of the activity of CYPs affected by polymorphic variants of POR, six full-length mutants of POR (e.g., Y181D, A287P, K49N, A115V, S244C and G413S) were designed and then co-expressed with CYP3A4 and CYP2B6 in the baculovirus-Sf9 insect cells to determine their kinetic parameters. Surprisingly, both mutants, Y181D and A287P in POR completely inhibited the CYP3A4 activity with testosterone, while the catalytic activity of CYP2B6 with bupropion was reduced to approximately ∼70% of wild-type activity by Y181D and A287P mutations. In addition, the mutant K49N of POR increased the CL*int* (V*max*/K*m*) of CYP3A4 up to more than 31% of wild-type, while it reduced the catalytic efficiency of CYP2B6 to 74% of wild-type. Moreover, CL*int* values of CYP3A4-POR (A115V, G413S) were increased up to 36% and 65% of wild-type respectively. However, there were no appreciable effects observed by the remaining two mutants of POR (i.e., A115V and G413S) on activities of CYP2B6. In conclusion, the extent to which the catalytic activities of CYP were altered did not only depend on the specific POR mutations but also on the isoforms of different CYP redox partners. Thereby, we proposed that the POR-mutant patients should be carefully monitored for the activity of CYP3A4 and CYP2B6 on the prescribed medication.

## Introduction

Members of cytochrome P450 monooxygenase superfamily (CYP) are known to catalyze the biotransformation of xenobiotics, including therapeutic drugs, carcinogens and many other environmental chemical compounds [1]. Cytochrome P450 3A4 (CYP3A4), the most abundant CYP isoform (up to 30% of total CYP content), had been identified in the liver and small intestine, and is known to play a significant role in the oxidation process of 40–50% therapeutic drugs [2], [3]. On the other hand, cytochrome P450 2B6 (CYP2B6) represents only 2%∼10% of total human hepatic complement of cytochrome P450 enzymes, which has been identified to participate in the metabolism of many clinically important drugs such as bupropion (antidepressant), cyclophosphamide (anticancer), efavirenz (antiretroviral), and propofol (anesthetic) [4].

Apparent variation of CYPs-mediated drug metabolism could alter therapeutic efficiency and toxicity of many drugs in clinical treatments, which was thought to be caused by CYPs polymorphisms in early studies [5], [6]. However, all the frequencies of identified CYPs polymorphisms can not completely explain the wide variation in CYPs-mediated drug metabolism [7]. In a clinical study, it has been shown that the variations in CYP3A4-mediated metabolism of midazolam in vivo did not correlate with the gene polymorphism of CYP3A4, but was found associated with cytochrome P450 oxidoreductase (POR) variant A503 V [8]. Further studies on functional characterization of the POR mutants on warfarin in vivo revealed that, in addition to several known factors contributing to the variations in warfarin maintenance dose (*VKORC1*_1639A>G, CYP2C9*2, CYP2C9*3, *CYP4F2* rs2108622, and chronic aspirin therapy), three common *POR* SNPs (−173C>A,−208C>T, and rs2868177) were also significantly associated with variations in warfarin maintenance dose [9]. Thus, it was suggested that CYP enzymes were not only the probable source of genetic element to cause metabolic variation, rather there may be some other factors such as certain functional partners of CYP and its gene polymorphisms that may influence the drug metabolism. Recently, few researchers have indicated that the polymorphic variants of cytochrome P450 oxidoreductase (POR) can influence the cytochrome P450 (CYP) mediated metabolism of drugs [7], [10], [11], [12]. Thereby, the mechanism underlying the altered drug metabolism by CYPs and its functional partners should be clearly identified.

POR is a flavoprotein with 680 amino acids (NCBI NP_000932.3) localized in the endoplasmic reticulum (ER) as the sole electron donor for all microsomal CYP enzymes, hemeoxygenase and fatty acid elongase [13], [14]. Moreover, it has been suggested that POR also participates in the metabolism of several chemical molecules such as mitomycin C, doxorubicin, cytochrome c, ferricyanide, menadione, dichlorophenolindophenol and nitro blue tetrazolium either directly or as an electron donor for these actual catalysts [15], [16]. Targeted disruption of the POR gene in mice providing a knockout (KO) animal model resulted in an early embryonic lethality [17], [18], while liver-specific knockout of POR in mice lead to a significant decrease of hepatic drug metabolic ability, and no morphological and reproductive differences were observed between the wild-type and liver-specific POR knockout mice [19], [20], suggesting that POR may also exert its key role in embryonic development in spite of its contributions for drug metabolism.

NADPH-cytochrome P450 oxidoreductase (POR) catalyzes the transfer of electrons from nicotinamide adenine dinucleotide phosphate (NADPH), via two flavin cofactors namely, flavin adenine dinucleotide (FAD) and flavin mononucleotide (FMN), to various cytochrome P450s and catalyzes the one-electron reduction of many drugs and xenobiotics [7]. On the other hand, the gene encoding human POR was found to be genetically polymorphic: 3.1 single nucleotide polymorphisms perkilobase were found among the 1682 alleles from 842 healthy people, and more than 40 naturally occurring POR variants in humans with changes of single amino acid have been identified [7], [21], [22]. Site-mutagenesis studies have clearly indicated that the mutations in the NADPH, FMN, FAD binding domains of POR inhibited the transfer of electron from NADPH to P450, and reduced the reduction ability of cytochrome c and CYP-mediated metabolism [23], [24], [25], [26], [27].

In fact, the majority of studies on POR variants were initially focused on an autosomal recessive genetic disease (formerly known as P450 oxidoreductase deficiency) with clinical phenotypes of ambiguous genitalia, congenital adrenal hyperplasia, skeletal malformation, Antley-Bixler syndrome (ABS), and polycystic ovary syndrome [28]. However, some recent studies have attempted to identify the possibility of POR as a potential rate-limiting step in CYP-mediated drug metabolism *in vitro* (reconstituted) systems, using N-terminal end-deleted CYPOR by bacterial system (prokaryocytes) since 2008.

Miller’s group have evaluated the impact of polymorphic variants of POR on the catalytic activity of CYP1A2 and CYP2C19 [29] as well as the effects of genetic variants of POR on catalytic activity and substrate-specific modulation of CYP3A4 and CYP2D6 in vitro, indicating that the activities of individual POR mutants might probably depend on the electron recipient and substrates [30], [31].

The present study was carried out to reveal the potential role of POR genetic variations on the CYP3A4 and CYP2B6 mediated metabolic activities and to help set up a database about the influence of POR genetic polymorphisms as a biomarker to predict the POR-involved metabolic activities of clinical drugs *in vitro*. In order to maintain the physiological catalytic activity of POR and CYP as practically as possible, we have selected the following six full-length POR missense mutations (e.g., K49N, POR*25; A115V, POR*11; Y181D, POR*8; S244C, A287P, POR*5; G413S). These mutations in POR (e.g., Y181D, A287P, A115V and G413) are associated with Antley-Bixler Syndrome, and A287P was found to be the most common POR mutation in the European population. Missense mutation K49N is located in the amino-terminal tail of POR which is responsible for anchoring POR into the endoplasmic reticulum (or plasma membrane) and is important for proper electro transfer function, similarly, S244C is located within the hinge close to FAD and FMN domains of POR. Therefore, we expressed these six POR missense mutations in baculovirus/Sf9 insect cell system (i.e., eukaryotic cell), and then co-expressed with wild-type human CYP3A4 and CYP2B6 respectively, to investigate the influence of these POR genetic variations on the metabolic activities of CYPs (i.e., CYP 3A4 and 2B6).

## Materials and Methods

### Materials

Polymerase chain reaction (PCR) primers were synthesized by Sunny Biotechnology (Shanghai, China). Restriction endonucleases, DNA molecular marker, and T4 ligase were obtained from MBI Fermentas (Amherst, NY). Site-directed Mutagenesis Kit (Stratagene, Cambridge, UK), Cellfectin reagent, pFastBac1 vector, DH10Bac-competent cells, Grace’s medium and fetal bovine serum were purchased from Invitrogen (Calsbad, CA). Spodoptera frugiperda Sf9 insect cells were obtained from Yangshengtang Company (Hainan, China). The pFastBAc-3A4 and 2B6 plasmids were kindly provided by Dr. Su Zeng (Zhejiang University). The primary anti- histidine antibody and Peroxidase-conjugated goat anti-mouse secondary antibody were purchased from Abmart (Shanghai, China) and Jackson ImmunoResearch Laboratories, Inc (Pennsylvania, USA) respectively. Testosterone and bupropion (chemical purity >99.0%) were purchased from National Institutes for Food and Drug Control (Beijing, China). Ammonium acetate, acetic acid, formic acid, acetonitrile, MgCl_2_, Tris–HCl and other chemicals and solvents were analytical reagents obtained from Sinopharm Chemical Regent Co. (Beijing, China) or chromatographic grade obtained from Tedia, Co. (Fairfield, OH, USA).

### Construction of Human POR Variant cDNAs

Site-directed mutagenesis was performed using Site-directed Mutagenesis Kit (Stratagene, Cambridge, UK) according to manufacturer’s instructions. The template plasmid with cDNAs encoding wild-type POR tagged with histidine (His6) has been previously cloned into pFastBac1 in our laboratory. Primers used in site-directed mutagenesis are listed in Table S1, and the mutated condos have been underlined. We amplified the DNA containing desired mutations by PCR, with initial denaturation at 94°C for 1 min followed by 32 cycles of denaturation at 94°C for 50 s, annealing at 60°C for 50 s, and extension at 72°C for 7 min 30 s. Final extension was done at 72°C for 7 min. After PCR amplification, the full-length cDNAs were completely sequenced to ensure that there were no extra mutations. The mutant POR cDNAs were then subcloned into the BamH I and Xho I sites of the donor vector pFastbac1. The names of six full-length POR missense mutations (i.e., K49N, POR*25; A115V, POR*11; Y181D, POR*8; S244C; A287P, POR*5; G413S) were classified according to the Human CYPallele nomenclature committee (http://www.cypalleles.ki.se/por.htm).

### Heterologous Expression of CYP3A4, CYP2B6 and POR Enzymes

The Bac-to-Bac baculovirus expression system was used for the expression of CYP3A4, CYP2B6 and POR enzymes. All steps for the production of recombinant proteins were carried out as described previously [32]. To obtain the reconstructed bacmid DNAs, each individual recombinant pFastbac-3A4, pFastBac-2B6 or the wild-type and mutant pFastBac-POR donor plasmids were separately transformed into competent DH10BAC cells, which contained the baculovirus DNA. The reconstructed bacmids were then transfected into Sf9 cells to obtain the recombinant baculovirus stock. All stocks were successively amplified to a final titer of approximately 1.0×10^8^ pfu/ml. Sf9 insect cell were obtained from Yangshengtang Company (Hainan, China) and maintained at 27°C in Grace’s medium containing 10% FBS. Sf9 cells were co-infected by CYP3A4 or CYP2B6 with POR recombinant baculovirus particles, and then Hemin stock solution (2 mg/ml, prepared by dissolving hemin in 50% ethanol and 0.2MNaOH) was added to the culture medium for a final concentration of 2 µg/ml. After incubation for 72 h, the infected Sf9 cells were harvested, washed with ice-cold phosphate balanced solution (PBS, pH7.4) and re-suspended in the lysis buffer (100 mM K_3_PO_4_,1 mM EDTA, 0.1 mM PMSF and 20% glycerol, pH7.4). The microsomes were prepared by centrifugation at 9000×g for 10 min at 4°C after sonication of cells to obtain the supernatant which was then stored at −80°C until use.

### Real-time PCR Analysis

The recombinant viral titers of CYP3A4, CYP2B6 and wild-type or mutant PORs were identified using real-time PCR, it has been previously demonstrated that there exists good agreement between DNA copy number and virus titers [33]. Total virus genome was extracted from 200 µl virus supernatant by Body Fluid Viral DNA/RNA Kit (Axygen, Hangzhou, China). Total of the viral DNA copy number was determined by quantification of viral DNA molecules by real-time PCR according to the manufacture’s instruction of SYBR Premix Ex Taq^Tm^ Kit (Takara, Dalian, China). The primers ATGATCAACATGGGAGACTCCCACGTGG (forward) and CTAGCTCCACACGTCCAGGGAGTAGC (reverse) were used to detect the viral DNA.

### Western Blot Analysis

Sf9 cells were harvested, and washed twice with cold PBS. Cell pellets were lysed by sonication in the presence of protease inhibitors and the total protein concentration was determined by BCA protein assay kit. Twenty microgram of each protein sample was resolved by 8% SDS-PAGE and electroblotted onto nitrocellulose membranes (Bio-Rad, Mississauga, ON). The membranes were blocked for 1 h at room temperature in PBS containing 5% skim milk plus 0.1% Tween-20 (PBST) and was incubated overnight at 4°C with monoclonal antibody against histidine as primary antibody (1∶3000 dilution), followed by incubation with a secondary antibody (goat anti-mouse IgG-HRP, 1∶5000 dilution) for 1 h at room temperature. The blots were detected with Immobilon Western chemiluminescent HRP (horseradish peroxidase) substrate (Millipore) by a lumino image analyzer, LAS-1000 plus (FujiFilm) according to the manufacturer's instructions. The amount of POR in each microsomal fraction was detected by western blotting, and the quantification of the immunoblots was performed by measuring the scanned band intensities using Quantity One-4.6.2 Software. [32]. In addition, the content of CYP3A4 and CYP2B6 co-expressed with PORs in the microsomal fractions of Sf9 cells was quantitated by CO-difference spectra using a molar extinction coefficient of 91 mM^−1^ [34].

### POR Activity Assay

POR activity assay was performed by the reduction of cytochrome c in the presence of wild-type POR and mutants POR respectively [35]. Each sample was assayed in 840 µL reductase assay buffer (50 mM potassium phosphate, 0.1 mM EDTA, 0.3 M KCl, pH7.4) supplemented with 100 µL of cytochrome c (0.4 mM), and 50 µg of POR protein in aerobic conditions at 30°C. Following incubation, the reaction was initiated by adding NADPH to the reaction mixture to obtain a final concentration of 5 mM. The rate of reduction of cytochrome c was monitored at 550 nm for 3 min against a blank sample containing the reductase assay buffer only.

### Enzymatic Activity of CYP3A4-POR and CYP2B6-POR

Microsomes of CYP3A4 and CYP2B6 co-expressed with wild-type or mutant PORs were determined for their metabolic ability for testosterone and bupropion respectively. Each sample was pre-incubated for 5 min at 37°C in a total volume of assay buffer (100 µL), which included 50 pmol of CYP3A4-POR or CYP2B6-POR microsomes, 100 mM Tris-HCl (pH7.4), 15 mM MgCl_2_, NADPH generating system resulting in a final concentration of 1 unit isocitrate dehydrogenase/ml and 5 mM isocitrate. Concentrations of testosterone or bupropion in the incubation mixtures ranged from 20 µM to 400 or 20 µM to 800 µM for CYP3A4-PORs or CYP2B6-PORs. The reaction mixture was started by addition of NADPH/NADP^+^ at final concentration of 1 mM and terminated after 60 min by the addition of two volumes of ice-cold acetonitrile. After incubation, the reaction mixture was centrifuged at 16,000×g for 20 min to obtain the supernatant and pellet protein. The supernatant (20 µL) was then assayed directly by using HPLC (Agilent Technologies 1200 series). All chromatographic separations were obtained using a Dikma Technologies’s, Diamonsil C18 column (5 µm, 200×4.6 mm). For detection of testosterone, it was monitored at 244 nm by C18 column with acetonitrile-water (53:47, v/v) as mobile phase, at a flow rate of 1.0 ml/min [36]. For detection of bupropion, it was monitored at 245 nm by C18 column with acetonitrile-KH_2_PO_4_ (0.01 mol/L, 0.1% triethylamine, pH 6.0) 40:60 (v/v) as mobile phase, at a flow rate of 1.0 ml/min as reported elsewhere [37]. All the mobile phases were vacuum filtered through a 0.22 µm membrane before use. The corresponding amount of metabolites at various concentrations of testosterone and bupropion were identified by external standard method. Because the pure standards of the hydroxytestosterone and hydroxybupropion were not commercially available, we used their substrates as the standard stock solution for the assay of their metabolites. The data was fitted with Michaelis-Menten model using GraphPad Prism 5 software (GraphPad Software, La Jolla, CA).

### Statistical Analysis

Each experiment was performed at least three times and all data has been presented as mean ± S.D. The Vmax and apparent Km values were determined using nonlinear regression analysis of Michaelis-Menten model (GraphPad Prism 5, Vision 5.01, GraphPad Software Inc.) Statistical analysis of data was carried out using a one-way ANOVA followed by Holm-Sidak pairwise multiple comparison test (Sigmaplot, Systat Software Inc). A probability value of less than 0.01 and 0.05 (*p<0.01 and *p<0.05) was accepted as a significant difference.

## Results

### Expression of the Recombinant CYP3A4-POR and CYP2B6-POR Proteins in sf9 Insect Cells

In order to better understand the effects of polymorphic variants of POR on CYPs, six different mutants POR were heterologously co-expressed with CYP2B6 and CYP3A4 in sf9 insect cells and were compared with activity of wild-type. Figures S1, S2, and S3 show the protein expression levels in sf9 insect cells after infection of various mutants of POR with CYP2B6 or CYP3A4. Immunoblot analysis clearly detected protein band at 78, 54 or 56 kDa for POR, CYP3A4 or CYP2B6 (respectively), and there were no appreciable differences observed among the protein expression levels of POR mutants, CYP3A4, CYP2B6 and wild-type, indicating all the different proteins were expressed uniformly by a site-directed mutagenesis. In addition, protein expression was not observed in the negative control group as shown in Fig.S1A-3A left panels (please see supplementary data).

The amount of POR in each microsomal fractions were quantitated by western blotting according to the published method [32], while the microsomal concentrations in sf9 cells of CYP3A4 or CYP2B6 co-expressed with PORs were quantitated by CO-induced difference spectra, as shown in Table 1 and Figure S4. In addition, we have also determined the viral titers of CYP3A4, CYP2B6, mutants POR and wild-type by real time-PCR, as shown in Table S2. In our preliminary experiment, we have found that the metabolic activities of CYP3A4 and CYP2B6 were much higher at the ratio of 2∶1 for CYPs to POR virus; thereby this ratio was selected for subsequent experiments.

**Table 1:** The content of CYP3A4 and CYP2B6 co-expressed with wild-type or six POR mutants in sf9 microsomal fractions were determined on the basis of reduced CO-difference spectrum.

Protein	CYP3A4 (pmol/mg protein)	CYP2B6 (pmol/mg protein)
WT	56.4±4.1	44.3±1.1
K49N	69.0±3.6	61.2±1.7
A115V	49.1±2.8	46.9±2.3
Y181D	74.1±6.7	49.2±4.5
S244C	48.3±2.0	36.8±1.8
A287P	59.5±3.3	54.5±3.8
G413S	43.9±2.1	56.9±1.9

### Enzymatic Activities of PORs

In order to understand the influences of mutations on POR activity, the specific enzymatic activities of six POR mutants were determined *in vitro* using reduction of Cytochrome c system, as described in method. The changes in the activity of each POR (i.e., six genetically-altered POR) in cells expressing PORs alone or co-expressing with CYP3A4 (or CYP2B6) are summarized in Table 2. Only a little amount of endogenous POR activity was found in the cells transfected with vector alone, while the enzyme activity (i.e., POR) was significantly increased by infection of external wild-type human POR in cells (Table 2). In addition, Sf9 cells infected with the virus of POR mutants (e.g., Y181D, A287P), showed more than ∼80% inhibition of POR activity in comparison to the wild-type. More interestingly, mutations by S244C and G413S variants enhanced the enzyme activity of wild-type up to approximately 13% and 77% (respectively). Regarding to the remaining two POR-mutants, namely, A115V and K49N, POR activity was decreased to 69–85% as compared to the wild-type activity, suggesting that the POR activities can be influenced by mutations in the amino acid sequences of POR.

**Table 2:** The reductive activity of wild-type and variant POR expressed alone and the activity of POR co-expressed with CYP3A4 or CYP2B6 in sf9 was estimated by NADPH-dependent Cytochrome c reduction.

Protein	POR (µmol/min/mg protein)		CYP3A4-POR (µmol/min/mg protein)		CYP2B6-POR (µmol/min/mg protein)	
Vector	0.04±0.002*	(1%)	–		–	
Wild-type	3.9±0.32#	(100%)	4.5±0.21	(100%)	4.7±0.15	(100%)
K49N	3.3±0.21#	(85%)	3.5±0.29	(78%)	4.5±0.25	(97%)
A115V	2.7±0.15# *	(69%)	3.1±0.13	(69%)*	4.3±0.37	(93%)
Y181D	0.5±0.07# *	(13%)	2.3±0.15	(50%)*	2.6±0.11	(56%)*
S244C	4.4±0.35#	(113%)	5.8±0.31	(130%)	5.3±0.18	(113%)
A287P	0.8±0.06# *	(21%)	2.5±0.13	(54%)*	2.9±0.43	(63%)*
G413S	6.9±0.54# *	(177%)	8.7±0.41	(193%)*	9.9±0.43	(211%)*

The activity of each POR mutant was calculated and expressed as a percentage of the activity of wild-type POR, arbitrarily set at 100%.

The values mean ±S.D. of three independent experiments.

# : *p*<0.01 in comparison with the vector control cells.

* : *p*<0.01 in comparison with the cells expressing wild-type (WT) POR.

On the other hand, the efficiency of each POR activity (e.g., activities of PORs were calculated and expressed as a percentage of the activity of wild-type POR) in cells co-expressing PORs with CYP3A4 (or CYP2B6) was found to be similar to the cells expressing PORs alone (Table 2), but activities of some of the POR mutants intended to increase in sf9 cells co-expressing PORs with CYP3A4 (or CYP2B6), suggesting that CYPs may alter the POR activity.

### Enzymatic Activities of CYP3A4-POR

**Figure 1:**
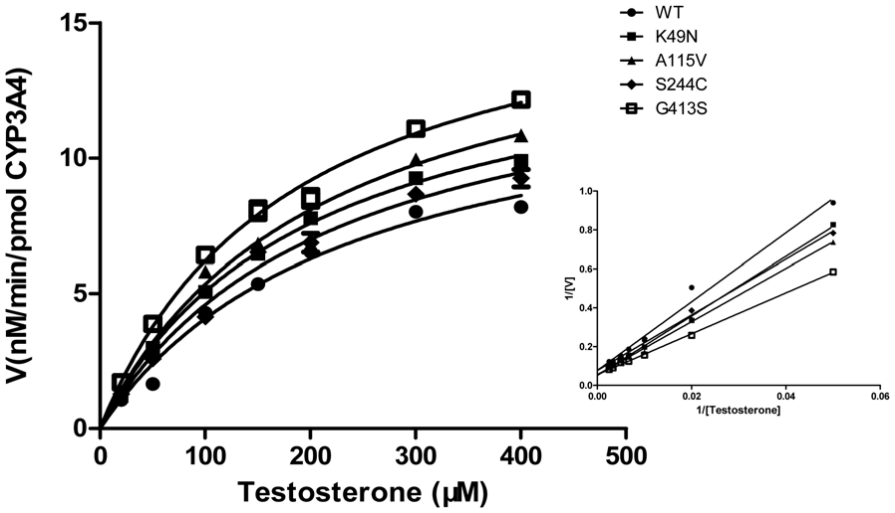
Determination of enzymatic activities of CYP3A4-PORs. Kinetics for the formation of hydroxytestosterone was determined by incubation of testosterone with CYP3A4–PORs, as described in method. Data are depicted as mean±S.D. (n = 3). The insert graphs show the Lineweaver–Burk plot of the data.

We further sought to determine whether the six polymorphic variants of POR can alter CYPs-mediated metabolic variations, for which CYP3A4 and CYP2B6 were co-expressed with six POR-mutants (respectively) in sf9 insect cells. Afterward, the metabolites of testosterone (as a specific substrate for CYP3A4) and bupropion (as a specific substrate for CYP2B6) were determined to establish the changes in the activities of CYP3A4 and CYP2B6. The metabolic products were determined by HPLC with their authentic standards (i.e., testosterone and bupropion). Figure 1 shows the metabolite of testosterone by CYP3A4 after being co-expressed with six different POR mutants and the kinetic parameters are summarized in Table 3. Surprisingly, some of the six polymorphic variants exhibited significantly altered activities of CYP3A4. In particular, activity of CYP3A4 was completely inhibited by the co-expression of Y181D and A287P (i.e., CYP3A4-POR (Y181D) and (A287P)) as in Table 3, and no metabolic products for testosterone (i.e., hydroxylation of testosterone) were found. These results strongly supported that CYPs functional partners may indeed influence the CYP-mediated metabolic activities.

**Table 3:** Km and Vmax values for CYP3A4 and CYP2B6 enzymes with different mutants of POR were determined by their specific substrates testosterone and bupropion.

POR	CYP3A4 - Testosterone				CYP2B6 – Bupropion				
	*K*m (nM/min/pmol)	*V*max (% of WT)		Cl*int* (μM)	*K*m (nM/min/pmol)		*V*max (% of WT)		Cl*int* (μM)
**WT**	236.7±10.3	13.70±0.88	0.058±0.0039 (100)		388.8±20.02	10.59±0.54		0.027±0.0027 (100)	
**K49N**	197.7±9.0*	15.08±0.31	0.076±0.0040 (131)*		311.1±12.13*	6.12±0.43*		0.020±0.0017 (74) #	
**A115V**	211.8±13.8	16.65±0.53*	0.079±0.0058 (136)*		294.6±26.19*	7.44±0.30*		0.025±0.0024 (93)	
**Y181D**	–	–	–		369.6.±10.96	7.10±0.26*		0.019±0.0015 (70) #	
**S244C**	219.7±22.6	14.65±0.74	0.067±0.0091 (115)		316.5±13.37*	9.95±0.44		0.031±0.0018 (115)	
**A287P**	–	–	–		475.1±24.91*	9.28±0.65		0.020±0.0015 (74) #	
**G413S**	183.3±13.3*	17.57±0.58*	0.096±0.0044(165)*		299.7±13.07*	6.65±0.36*		0.022±0.0019 (81)	

The ratio of Vmax to Km was used as an index of catalytic efficiency; the activity of each POR mutant co-expressed with CYPs was calculated and expressed as a percentage of the activity of wild-type POR, arbitrarily set at 100%.

The data were represented as mean ± S.D. of three independent experiments.

CL*int = V*max/*K*m.

* : *p*<0.01 in comparison with the cells expressing wild-type (WT) POR.

# : *p*<0.05 in comparison with the cells expressing wild-type (WT) POR.

Dash (–) indicates not detectable.

On contrary, the remaining three variants, K49N, A115V and G413S of POR showed a significant increase in the catalytic efficiencies of CYP3A4-POR, which was judged by the ratios of Vmax/Km (CL*int*) when compared with wild-type. In particular, CYP3A4-POR (G413S) exhibited the highest changes in the *K*m and *V*max (1.3-folds) values compared to wild-type, and increased the catalytic efficiency (CL*int*) up to 65% of wild-type. Moreover, CL*int* values of CYP3A4-POR (K49N) and CYP3A4-POR (A115V) were calculated to increase up to 31% and 36% of wild-type. Additionally, mutant S244C of POR also increased the activity of CYP3A4, but it was much less than that of K49N, A115V and G413S, as shown in Table 3.

### Enzymatic Activities of CYP2B6-POR

**Figure 2:**
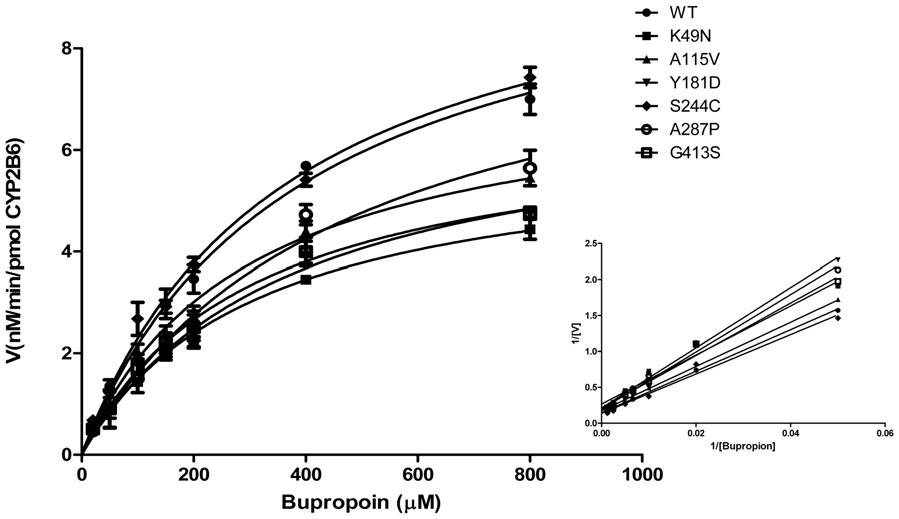
Determination of enzymatic activities of CYP2B6-PORs. Kinetics for the formation of hydroxybupropion was determined by incubation of bupropion with CYP2B6-PORs, as described in Method. Data are depicted as mean±S.D. (n = 3). The insert graphs show the Lineweaver–Burk plot of the data.

Regarding the impact of six POR genetic mutations on catalytic activities of CYP2B6 (i.e., CYP2B6-POR) with bupropion (as a specific substrate for CYP2B6), we found that the influences of POR genetic mutations on catalytic activities of CYP2B6 were different as compared to that of CYP3A4, as shown in Figure 2. More interestingly, regarding to the metabolite of bupropion by CYP2B6 in sf9 cells co-expressed with K49N (i.e., CYP2B6-POR (K49N)), the Vmax was significantly reduced to 58% of the wild-type, and the catalytic efficiencies were also reduced approximately up till 30%, while mutant K49N (i.e., CYP3A4-POR (K49N)) enhanced the catalytic efficiency of CYP3A4 up to 31% of wild-type, as shown in Figure 2 and Table 3.

With respect to A115V and G413S, the activities of CYP2B6 were not affected significantly by co-expressing with these two mutants (Table 3), while they increased the activity of CYP3A4 significantly as described above. In addition, although Y181D and A287P reduced the activities of CYP2B6 to approximately 70% of wild-type, however, activities of CYP3A4 were completely inhibited by the two mutants (Table 3).

## Discussion

Pharmacogenetics has been used recently to establish correlations between genetic polymorphisms and variations in drug efficacy, which might contribute to the development of personalized medicine in the near future. Additionally, an individual’s genetic makeup and genetic polymorphism may lead to optimization of therapeutic strategies resulting in maximum therapeutic efficiency and minimal adverse drug reactions [38], [39].

Cytochrome P450 oxidoreductase (POR) is known to play a critical role in delivering electrons to all microsomal P450 enzymes and many of the redox system [10]. Moreover, animal experiments have also indicated that the POR may contribute to the embryonic development, because the POR knockdown resulted in an early embryonic lethality. Recently, it has been reported that the genetic polymorphisms of POR had definitely affected endocrinology, pharmacology and as well as the developmental biology [17], [18]. Thus, the function and activities of POR (including genetic polymorphisms) should be clearly identified.

The human POR variants were recently expressed by using truncated cDNAs in bacterial system (prokaryocytes) to evaluate the effects of the POR on drug metabolism. However, in this system the expressed POR proteins were found to lack many amino acid residues (for example, 27 or 46 N-terminal amino acid residues) [29], [30], [31], [35], [40], [41], [42]. Recent studies have confirmed that the hydrophobic N-terminal sequence of NADPH cytochrome P450 reductase (residues 1–46) not only participate directly in orientation in the endoplasmic reticulum but also play a key role in cytochrome P450 interaction. It has been recently reported that the substantial differences were observed in the electron-transfer abilities between the full-length POR variants and N (27)-deleted form in *E.coli*, suggesting that the full-length POR variants may be more suitable for evaluating the human POR-variants [26], [43], [44]. In the present study, the wild-type and six polymorphic variants of POR with full-length gene were co-expressed in Sf9 insect cells (i.e., eukaryotic cell), which presented to be a more suitable physiological model for the evaluation of human variants. In order to ensure the amounts of viral titer co-added in Sf9 insect cells to be almost identical, we quantified each virus DNA copy number of six human genetic variations of POR (i.e., A115V, Y181D, K49N, S244C, A287P and G413S) or CYP3A4/CYP2B6 by real-time PCR (Table S2). Furthermore, the amount of POR in each microsomal fraction was quantitated by western blotting, while the contents of CYP3A4 and CYP2B6 in the microsomal fractions of Sf9 cells were quantitated by CO-difference spectra (Figs. S1, S2, S3, and S4).

In order to assess the activities of POR variants, each POR was expressed independently or co-expressed with CYP3A4 or CYP2B6 in sf9 cells. The activities of POR variants were determined by measuring reduction of Cyt c. Interestingly, the mutants Y181D and A287P in POR strongly reduced the activity to approximately 20% of wild-type, while S244C and G413S enhanced the POR activity up to 13% and 77% respectively compared with wild-type (Table 2), suggesting that the POR mutation could influence the original activity of wild-type. In addition, Y181D and A287P reduced the activity up to approximately 50% of the wild-type in cell co-expressing PORs with CYPs, and activities were also significantly increased when compared with cells expressing PORs alone (Table 2). This disparity in the results may be probably due to the protein-protein interactions between two different approaches, and further work is needed to confirm this conclusion.

Additionally, we determined the Km and Vmax values for two specific substrates (i.e., testosterone and bupropion) by CYP3A4-POR and CYP2B6-POR. The activity of CYP3A4 was completely inhibited after co-expressing with Y181D and A287P when compared with the wild-type (Table 3), this result was also found to be consistent with the early study where POR variant, Y181D located in FMN binding domain resulted in inhibition of CYP3A4 activity in prokaryotic expression systems [40], [45]. Similar results were also observed in P450 based assay, where the activities of CYPs (e.g., CYP17A1, CYP2C19, CYP1A2) were completely inhibited by mutation of FMN binding domain at POR (i.e., Y181D) [29], [46], [47], [48]. However, in the present study, differently from the former observations, the activity of CYP2B6 was not affected by POR mutants (i.e., Y181D and A287P) (Fig.2 and Table 3). Dhir et al (2007) reported that A287P of POR showed significantly lower activities in supporting 17, 20 lyase, CYP1A2 and CYP2C19, and had similar activities as compared to wild-type for CYP19A1 and CYP21A2, suggesting that A287P mutation may show variable effects when co-working with different redox partners [31], [47], [48]. Flück et al. (2010) has reported that 60% of CYP3A4 activity was lost by A287P mutant of POR with BOMCC, but the CYP3A4 activity on testosterone was completely lost by A287P mutant in our current study, suggesting that the impact of POR variants on the catalytic activity of CYP3A4 is substrate-specific.

Regarding the K49N mutation which was localized in transmembrane domain, the variation of CYP3A4-POR (K49N) for *V*max/*K*m values were increased up to 31% of wild-type, while it decreased in CYP2B6-POR (K49N) to 74% of wild-type (Table 3). Furthermore, POR mutant A115V and G413S enhanced the catalytic activities of CYP3A4 to 36% and 65% (respectively) despite of the metabolic activity of CYP2B6 to bupropion which was not significantly influenced by these two mutants (Table 3). S244C mutant is located in a highly flexible hinge region which is the connecting domain combining FAD and FMN together to facilitate the electron transfer. In fact, S244C resulted in increasing the catalytic efficiency of CYP3A4 and CYP2B6 (approximately 15%) in the present study, but according to other literature it reduced the catalytic efficiency of CYP17A1 and CYP21A2 up to more than 70% [47]. Regarding to the above information, it can be revealed that the same mutant in POR is able to make diverse impacts on the catalytic efficiency when co-working with different CYP redox partners.

To date, more than 50 microsomal P450 enzymes and 40 mutants POR have been found in human [7], [21], [22]. Therefore, each POR variants will require testing with each CYP superfamily member and other drug metabolizing P450s to assess their effects. To make the CYP-mediated drug metabolism affected by POR mutations more clearly, additional studies covering different other types of CYPs are required to take either *in vitro* or *in vivo* drug clearance tests. In summary, we suggested that the POR-mutant patients should be carefully monitored for the activity of CYP3A4 and CYP2B6 for the prescribed medication to obtain maximum therapeutic efficiency and minimal adverse drug reactions. Therefore, our present study would be helpful to set up a database about the influence of POR genetic polymorphisms as a biomarker to predict the POR-involved metabolic activity of clinical drugs.

## Supporting Information

Figure S1 **Immunoblot analysis of wild type and six mutant POR microsomal proteins in Sf9 cells.** (A) The position of POR proteins was detected at 78 kDa by Western blot. N: negative. W: wild type. (B) The relative expression levels of wild type and mutants POR in Sf9cells were quantified with Quantity One Software. The results are indicated as mean ± S.D. of three independent experiments.
(TIF)

Figure S2 **Immunoblot analysis of CYP3A4 microsomal proteins co-expressed with wild type or six mutants POR in Sf9 cells.** (A) The position of CYP3A4 proteins were detected at 54 kDa by Western blot after infections with wild type or mutants POR, as described in Methods. (-): negative. WT: wild type. (B) The relative expression levels of CYP3A4 proteins in POR-infected Sf9 cells were quantified with Quantity One Software. The results are indicated as mean ± S.D. of three independent experiments.
(TIF)

Figure S3 **Immunoblot analysis of CYP2B6 microsomal proteins co-expressed with wild type or six mutants PORsSf9 cells.** (A) The position of CYP2B6 proteins was detected at 56 kDa by Western blot after infections with wild type or mutants POR, as described in Methods. (-): negative. WT: wild type. (B) The relative expression levels of CYP2B6 proteins in POR-infected Sf9 cells were quantified with Quantity One Software. The results are indicated as mean ± S.D. of three independent experiments.
(TIF)

Figure S4 **CO-difference spectrum of the expressed CYP3A4 (A) and CYP2B6 (B).** Content was determined from the carbon monoxide difference spectrum and the molar absorption coefficient for cytochrome P450.
(TIF)

Table S1 **PCR primers used for site-directed mutagenesis of POR cDNAs.**
(DOCX)

Table S2 **Real-time PCR titration results for CYP3A4, CYP2B6 and wild type or mutant PORs.**
(DOCX)
