# Supplementary figures and images for: Influence of Various Polymorphic Variants of Cytochrome P450 Oxidoreductase (POR) on Drug Metabolic Activity of CYP3A4 and CYP2B6
Source: PLoS One. 2012 Jun 12;7(6):e38495. doi: 10.1371/journal.pone.0038495 (PMC3373556; doi:10.1371/journal.pone.0038495)

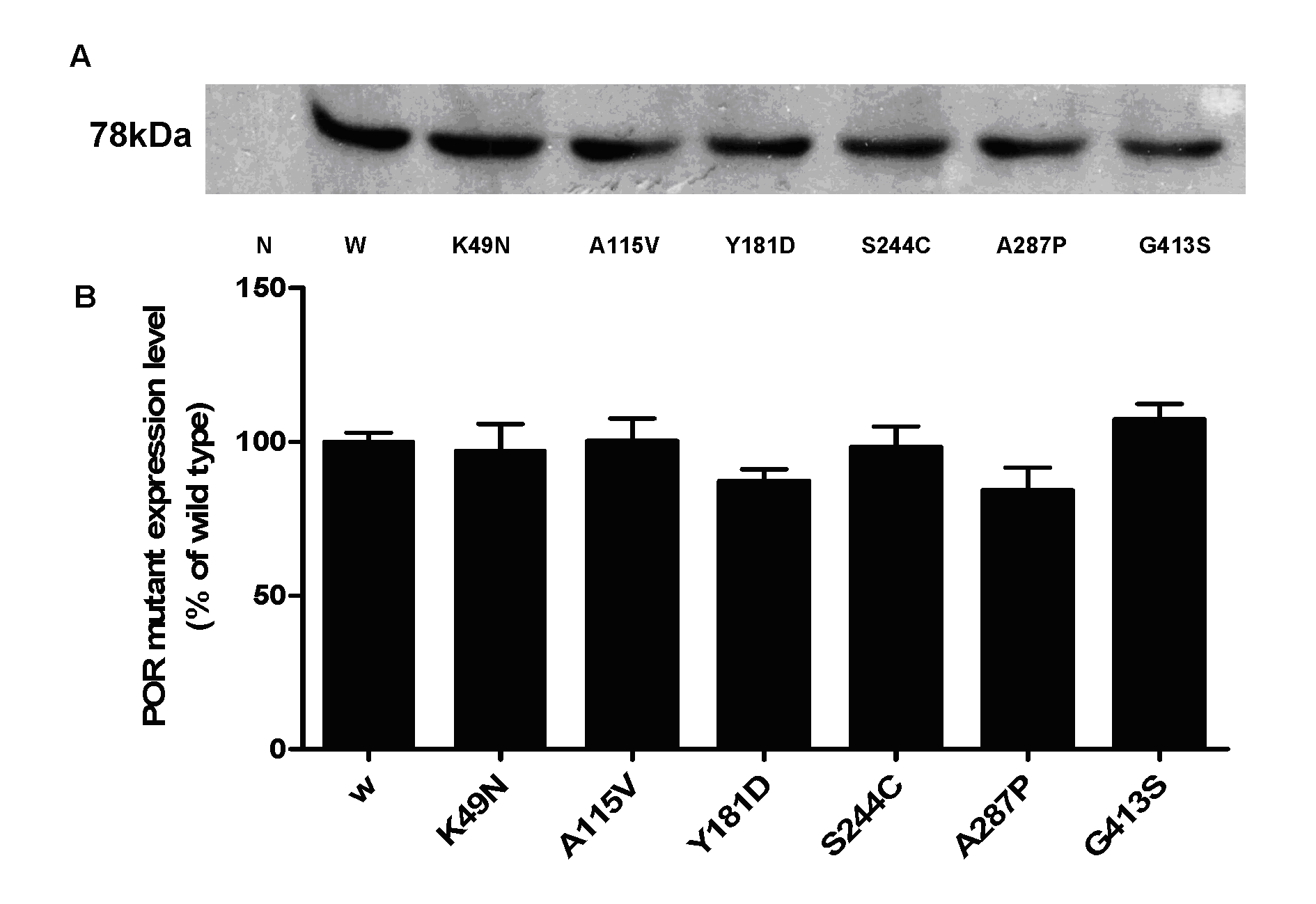

Supplement: Figure S1 — Immunoblot analysis of wild type and six mutant POR microsomal proteins in Sf9 cells. (A) The position of POR proteins was detected at 78 kDa by Western blot. N: negative. W: wild type. (B) The relative expression levels of wild type and mutants POR in Sf9cells were quantified with Quantity One Software. The results are indicated as mean ± S.D. of three independent experiments. (TIF) [file pone.0038495.s001.tif]

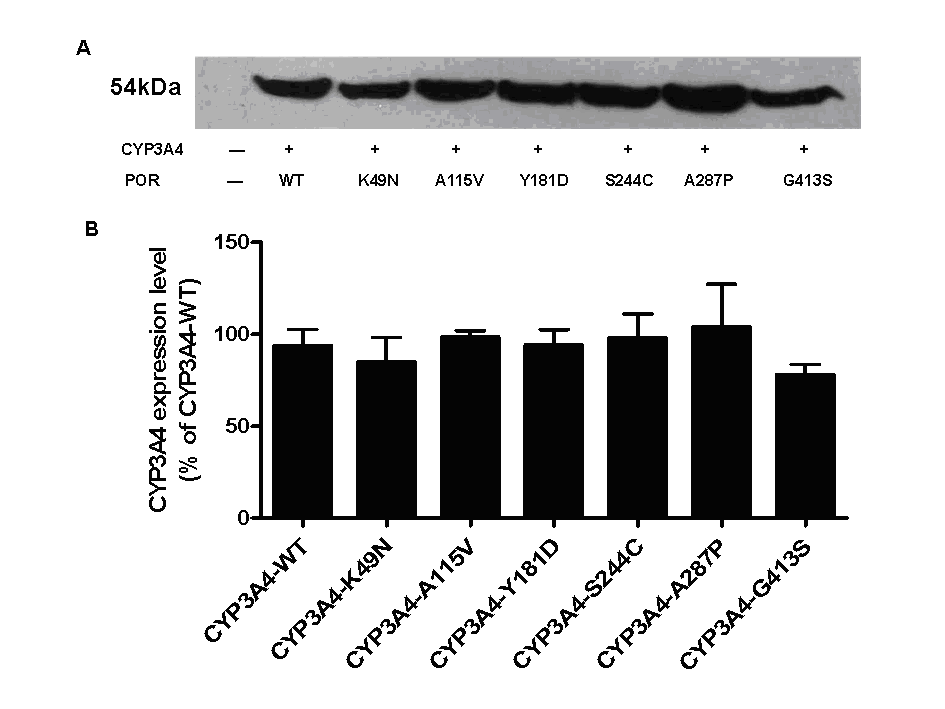

Supplement: Figure S2 — Immunoblot analysis of CYP3A4 microsomal proteins co-expressed with wild type or six mutants POR in Sf9 cells. (A) The position of CYP3A4 proteins were detected at 54 kDa by Western blot after infections with wild type or mutants POR, as described in Methods. (-): negative. WT: wild type. (B) The relative expression levels of CYP3A4 proteins in POR-infected Sf9 cells were quantified with Quantity One Software. The results are indicated as mean ± S.D. of three independent experiments. (TIF) [file pone.0038495.s002.tif]

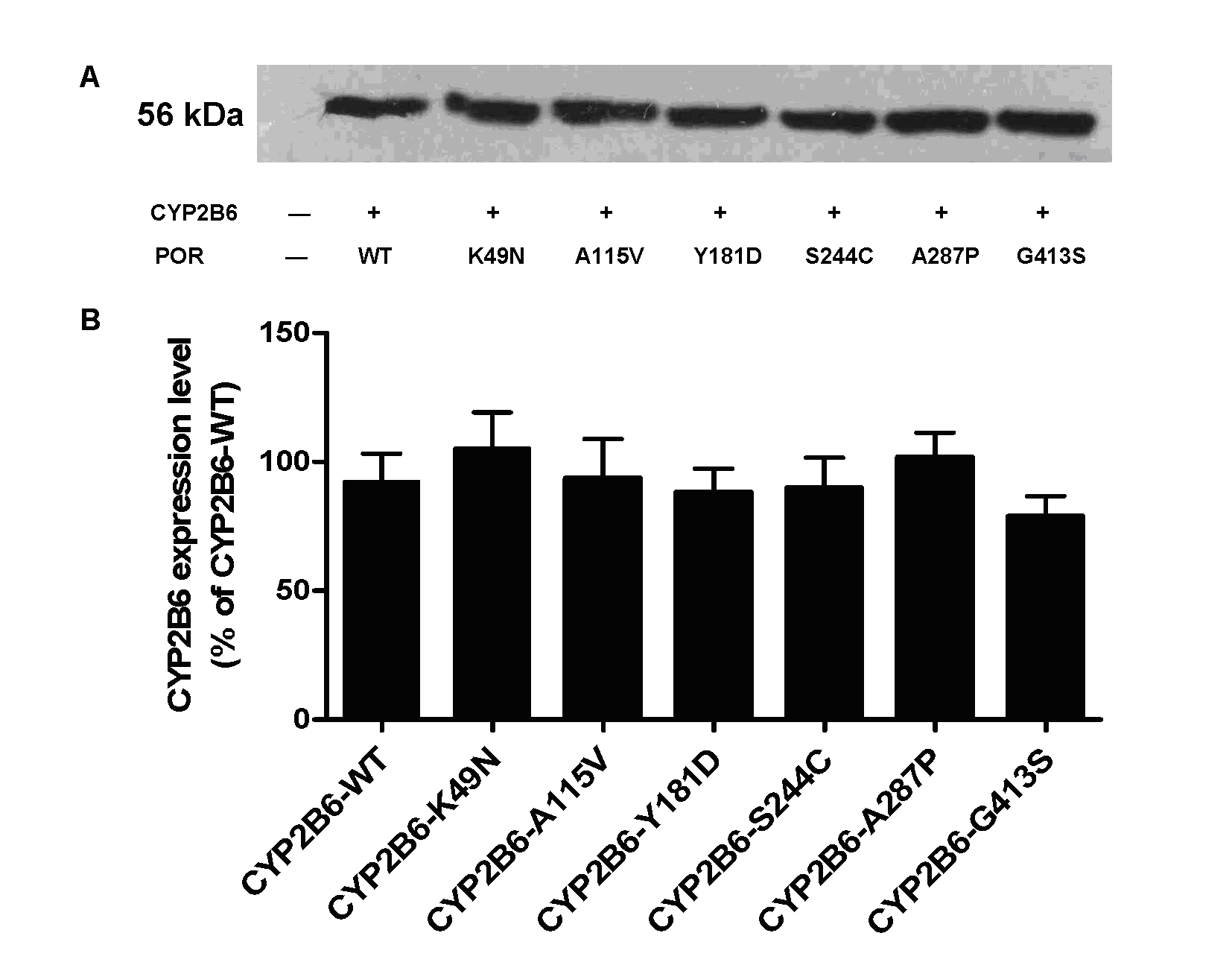

Supplement: Figure S3 — Immunoblot analysis of CYP2B6 microsomal proteins co-expressed with wild type or six mutants PORsSf9 cells. (A) The position of CYP2B6 proteins was detected at 56 kDa by Western blot after infections with wild type or mutants POR, as described in Methods. (-): negative. WT: wild type. (B) The relative expression levels of CYP2B6 proteins in POR-infected Sf9 cells were quantified with Quantity One Software. The results are indicated as mean ± S.D. of three independent experiments. (TIF) [file pone.0038495.s003.tif]

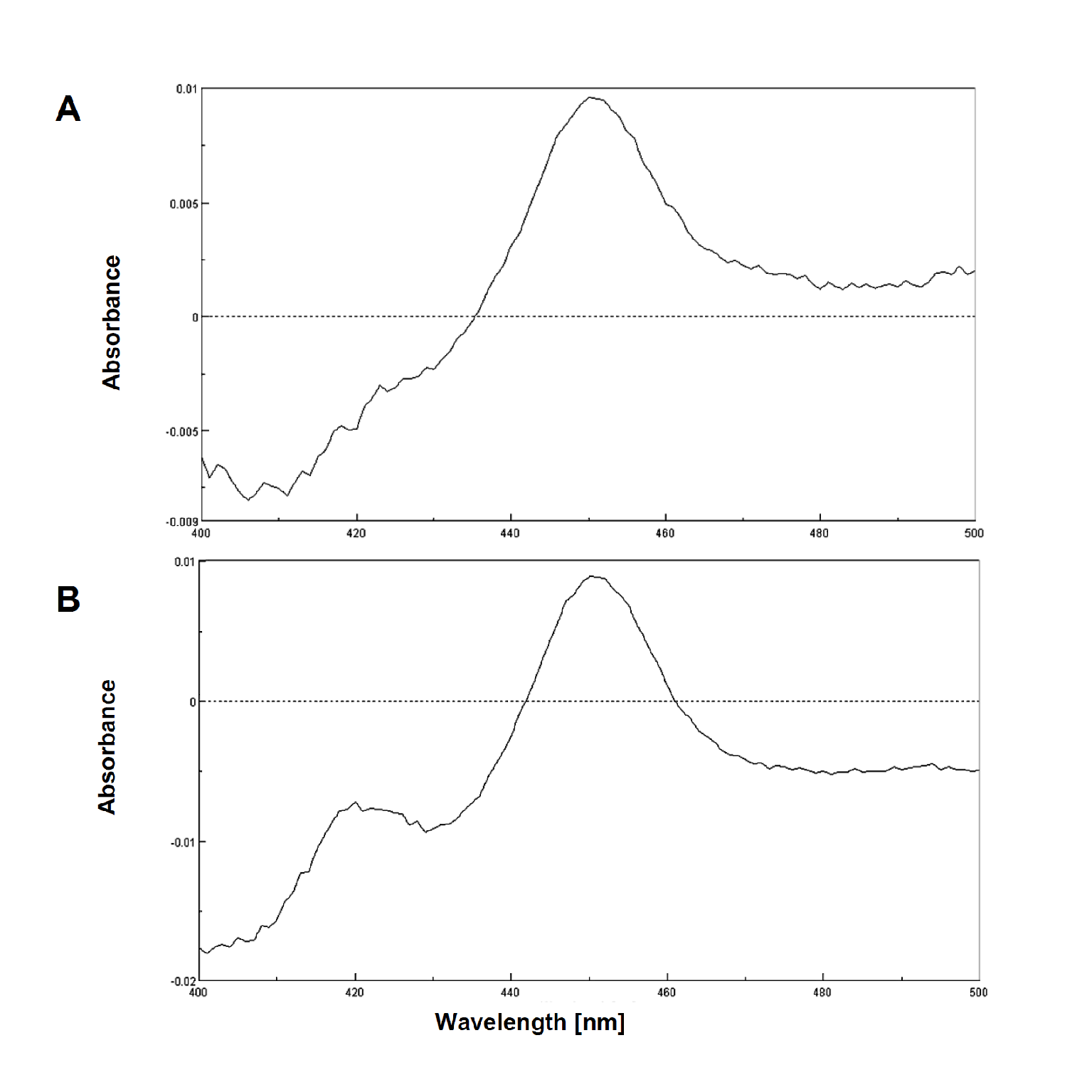

Supplement: Figure S4 — CO-difference spectrum of the expressed CYP3A4 (A) and CYP2B6 (B). Content was determined from the carbon monoxide difference spectrum and the molar absorption coefficient for cytochrome P450. (TIF) [file pone.0038495.s004.tif]
