# Supplementary material for: Influence of Various Polymorphic Variants of Cytochrome P450 Oxidoreductase (POR) on Drug Metabolic Activity of CYP3A4 and CYP2B6
Source: PLoS One. 2012 Jun 12;7(6):e38495. doi: 10.1371/journal.pone.0038495 (PMC3373556; doi:10.1371/journal.pone.0038495)
Supplement: Table S1 — PCR primers used for site-directed mutagenesis of POR cDNAs. (DOCX) [file pone.0038495.s005.docx]

**Table S-1.** PCR primers used for site-directed mutagenesis of POR cDNAs

| Mutations | Primers(F, forward; R, reverse) |
| --- | --- |
| K49N | **F:** ttcctcttcagaaaCaaaaaagaagaagtc  **R:** gacttcttcttttttGtttctgaagaggaa |
| A115V | **F:** gaggcatgtcagTggaccctgagg  **R:** cctcagggtccActgacatgcctc |
| Y181D | **F:** ggaacaagaccGacgagcacttc  **R:** gaagtgctcgtCggtcttgttcc |
| S244C | **F:** ggcgaggagtccTgcattcgccagtac  **R:** gtactggcgaatgcAggactcctcgcc |
| A287P | **F:** gaatccgttcctgCctgcagtcaccac  **R:** gtggtgactgcagGcaggaacggattc |
| G413S | **F:** cctcctcctccAgcgagggcaaggagc  **R:** gctccttgccctcgcTggaggaggagg |
