# Supplementary material for: Influence of Various Polymorphic Variants of Cytochrome P450 Oxidoreductase (POR) on Drug Metabolic Activity of CYP3A4 and CYP2B6
Source: PLoS One. 2012 Jun 12;7(6):e38495. doi: 10.1371/journal.pone.0038495 (PMC3373556; doi:10.1371/journal.pone.0038495)
Supplement: Table S2 — Real-time PCR titration results for CYP3A4, CYP2B6 and wild type or mutant PORs. (DOCX) [file pone.0038495.s006.docx]

**Table S-2.** Real-time PCR titration results for CYP3A4, CYP2B6 and wild type or mutant PORs.

| Virus gene | The Ct-values viral titer×10^8^ (pfu/mL) |
| --- | --- |
| Wildtype | 18.33±0.33 1.84±0.11 |
| K49N | 18.36±0.57 1.83±0.19 |
| A115V | 18.20±0.42 1.89±0.14 |
| Y181D | 18.70±0.22 1.72±0.07 |
| S244C | 17.86±0.61 2.00±0.20 |
| A287P | 17.43±0.52 2.14±0.17 |
| G413S | 18.45±0.09 1.80±0.03 |
| CYP3A4 | 17.83±0.12 2.00±0.09 |
| CYP2B6 | 17.38±0.28 2.13±0.09 |
